# Sexual orientation and symptoms of common mental disorder or low wellbeing: combined meta-analysis of 12 UK population health surveys

**DOI:** 10.1186/s12888-016-0767-z

**Published:** 2016-03-24

**Authors:** Joanna Semlyen, Michael King, Justin Varney, Gareth Hagger-Johnson

**Affiliations:** Department of Psychology, London Metropolitan University, London, UK; Division of Psychiatry, UCL, London, UK; Public Health England, London, UK; Administrative Data Research Centre for England (ADRC-E), Farr Institute, UCL, London, NW1 2DA UK

**Keywords:** Anxiety disorders, Depression, Health surveys, Homosexuality, Mental disorders, Meta-analysis, Mood disorders, Neurotic disorders, Sexual orientation, Sexuality

## Abstract

**Background:**

Previous studies have indicated increased risk of mental disorder symptoms, suicide and substance misuse in lesbian, gay and bisexual (LGB) adults, compared to heterosexual adults. Our aims were to determine an estimate of the association between sexual orientation identity and poor mental health and wellbeing among adults from 12 population surveys in the UK, and to consider whether effects differed for specific subgroups of the population.

**Methods:**

Individual data were pooled from the British Cohort Study 2012, Health Survey for England 2011, 2012 and 2013, Scottish Health Survey 2008 to 2013, Longitudinal Study of Young People in England 2009/10 and Understanding Society 2011/12. Individual participant meta-analysis was used to pool estimates from each study, allowing for between-study variation.

**Results:**

Of 94,818 participants, 1.1 % identified as lesbian/gay, 0.9 % as bisexual, 0.8 % as ‘other’ and 97.2 % as heterosexual. Adjusting for a range of covariates, adults who identified as lesbian/gay had higher prevalence of common mental disorder when compared to heterosexuals, but the association was different in different age groups: apparent for those under 35 (OR = 1.78, 95 % CI 1.40, 2.26), weaker at age 35–54.9 (OR = 1.42, 95 % CI 1.10, 1.84), but strongest at age 55+ (OR = 2.06, 95 % CI 1.29, 3.31). These effects were stronger for bisexual adults, similar for those identifying as ‘other’, and similar for 'low wellbeing'.

**Conclusions:**

In the UK, LGB adults have higher prevalence of poor mental health and low wellbeing when compared to heterosexuals, particularly younger and older LGB adults. Sexual orientation identity should be measured routinely in all health studies and in administrative data in the UK in order to influence national and local policy development and service delivery. These results reiterate the need for local government, NHS providers and public health policy makers to consider how to address inequalities in mental health among these minority groups.

**Electronic supplementary material:**

The online version of this article (doi:10.1186/s12888-016-0767-z) contains supplementary material, which is available to authorized users.

## Background

Around 1–2 % of the United Kingdom’s adult population identify as lesbian, gay or bisexual (LGB) [1, 2] and 5 % as non-heterosexual [2], although because sexual orientation comprises identity, behaviour and attraction [3], the chosen definition used can lead to variability in these estimates. We know that sexual minority populations experience poorer physical heath [4] and engage in riskier health behaviours such as smoking and hazardous drinking [5]. These inequalities may emerge in adolescence and early adulthood, then persist throughout the lifecourse [6].

Symptoms of poor mental health (e.g. anxiety, depression) and low wellbeing (e.g. not having ‘positive mental health’ [7]) are common in the adult population but there is established evidence that adults who identify as lesbian, gay or bisexual are at higher risk of experiencing these symptoms than adults who identify as heterosexual. A systematic review of the prevalence of mental disorder, substance abuse, suicidality and self-harm in LGB people showed that these populations experience a greater incidence of depression, anxiety, suicidality and substance misuse [8] than heterosexuals. Meta-analysis following this review found that LGB people were around twice as likely to have attempted suicide in their lifetime and have around 1.5 times higher prevalence of depression and anxiety disorders in the preceding 12 months. Associations between minority sexual orientation and poorer mental health have persisted over time with recent studies showing the same effects as older studies [9]. Such disparities are thought to emerge early in adolescence and persist into adulthood [10].

Population-based evidence of poorer mental health in LGB people has been found in samples from the United States [11, 12], Netherlands [9] and England [13]. Although these findings are restricted to high-income countries, data from low income countries are minimally available [14]. Population surveys involving comprehensive psychiatric interviews have shown increased risk of common mental disorders among LGB adults [2, 13]. Similar results have been found in clinical, community and convenience samples [13, 15–17]. Until recently however, sexual orientation was not recorded routinely in UK population health surveys [5] and remains poorly recorded in national data collection from services such as the UK’s Increased Access to Psychological Therapies (IAPT) services. This lack of recording of sexual orientation presents challenges when targeted or tailoring interventions to LGB adults and in particular, subgroups of LGB adults with specific service needs [18, 19]. Few studies have considered which subgroups of the LGB adult population are most at risk of poor mental health (for example, by age group, sex, ethnic minority status or educational attainment). The aim of our study was therefore to evaluate whether the association between sexual orientation identity and mental health (common mental disorder and low wellbeing) was different in different subgroups of the non-heterosexual identified adult population in the United Kingdom, using newly available pooled data from 12 population health surveys.

## Method

### Design and setting

Participants were from the British Cohort Study (BCS) 2012 [20], Health Survey for England (HSE) 2011 [21], 2012 [22] and 2013 [23], Scottish Health Survey (SHS) 2008 to 2013 [24], the Longitudinal Study of Young People in England (LSYPE) 2009/10 [5] and Understanding Society (US) 2011/12 [25], identified by searching the UK Data Service (search terms ‘sexual orientation’, ‘gay’, ‘lesbian’, ‘bisexual’, ‘sexuality’). All studies collected data using either home visit interviews, self-completion questionnaires, telephone interviews, web surveys or a combination (details including sampling designs and inclusion/exclusion criteria are available from the UK Data Archive). Our study population comprised adults with available data on sexual orientation identity, symptoms of mental health and wellbeing, and study covariates.

### Participants and materials

Participants: For all studies included in our analysis, participants were recruited through random or stratified random sampling of their target population.

#### Sexual orientation identity

Sexual orientation identity was recorded in self-completion questionnaires in all included studies using standardised wording recommended by the Office of National Statistics (ONS) [1]. Participants were asked ‘Which of the following options best describes how you think of yourself?’ Response options were ‘Heterosexual or Straight’, ‘Gay or Lesbian’, ‘Bisexual’, ‘Other’, or refusal. Participants who refused to answer this question were excluded from our analysis, on the basis that they could not be assigned to any sexual orientation identity category. Table 1 shows the refusal rate across all studies.

**Table 1:** Characteristics of study variables

Total *n* = 94,818	British Cohort Study	Health Survey for England			LSYPE	Scottish Health Survey						Understanding Society
Year sexual orientation identity recorded	2012	2011	2012	2013	2009/10	2008	2009	2010	2011	2012	2013	2011/12
Study sample size	6,958	7,116	7,033	7,299	6,310	5,091	6,208	6,229	6,391	4,149	4,320	27,714
Refused identity item^a^ (%)	0.5	3.0	3.0	2.0	0.4	6.5	4.6	5.2	4.4	2.9	3.1	0.1
GHQ score ≥4 (%)	-	-	15.1	-	22.3	14.3	14.4	15.9	15.0	14.7	15.1	18.9
Anxious/depressed (%)	-	26.5	19.8	-	-	-	-	-	-	-	-	-
Low wellbeing score (%)	31.1	21.6	19.5	21.7	-	27.9	28.6	30.4	27.7	28.4	27.8	-
Age (range 16;103)	40;40	16;96	16;98	16;104	16;19	18;93	18;101	18;96	18;103	18;99	18;99	16;100
Male (%)	45.8	44.6	44.7	44.6	49.6	43.6	43.9	42.9	43.0	44.1	43.1	43.3
Lesbian/Gay	1.9	0.9	1.0	1.3	1.3	0.7	0.8	0.9	0.8	0.9	0.8	1.3
Bisexual	0.6	0.6	0.7	0.7	1.7	0.9	0.8	1.0	1.0	0.5	0.6	1.1
‘Other’	0.2	0.6	0.4	0.6	0.2	0.8	0.6	1.4	1.4	0.7	0.8	1.1
Ethnic minority^b^	3.1	9.4	9.3	10.0	30.1	2.4	2.1	2.8	2.5	2.6	2.6	13.1
University degree	24.9	25.1	26.1	26.5	18.2	26.9	27.6	28.5	28.0	30.0	30.7	24.2
Smoker	23.3	19.9	18.8	19.2	20.8	39.4	36.0	36.4	36.1	38.8	38.3	55.1
Longstanding illness	14.8	42.4	41.2	41.3	6.6	43.0	42.0	47.7	47.1	34.3	33.4	34.1
Married/co-habiting	80.7	64.2	64.2	54.2	30.7	67.4	67.0	63.5	65.5	65.5	63.4	63.1

Note. ^a^Refusal to answer sexual orientation identity item (excluded from the analytic sample).^b^LSYPE and Understanding Society over-sample ethnic minority groups, explaining the high proportions seen here

#### Symptoms of common mental disorder

Meeting the threshold for symptoms of mental health was defined as scoring 4 or higher on the General Health Questionnaire-12 (GHQ-12) [22, 26]. For HSE, cases were defined as moderately/severely depressed on the EQ-5D subscale which measures very similar mental health symptoms [22, 27–29]. Both instruments have been validated for the detection of clinically significant symptoms of mental disorder [7, 26, 30, 31]. Sensitivity analyses (described below) evaluated whether differences between these instruments influenced the results.

#### Low wellbeing score

Low wellbeing was defined as falling in the lowest sex-specific quartile of scores on the Warwick-Edinburgh Mental Wellbeing Scale (WEMWBS) [7] in the analytic sample for our study, on the basis that there is currently no established threshold for defining ‘low wellbeing’ in the adult population. The WEMWBS has been validated as a measure of ‘positive mental health’ which covers a wider range of concepts than the absence of mental disorder, including the subjective experience of happiness and life satisfaction, psychological functioning and self-realisation.

#### Covariates

Covariates were selected on the basis that they are known to be associated with sexual orientation identity and with symptoms of mental health and wellbeing (i.e. are potential confounding factors). Covariates were harmonised across studies to ensure comparability: sex (male or female), ethnic group (White vs. ethnic minority), educational attainment (a five-point scale ranging from ‘none’ to University degree), smoking status (current vs. ever smoker), longstanding illness/disability (yes or no) and married or co-habiting (yes or no).

### Statistical analysis

Bivariate associations between sexual orientation identity and mental health were evaluated using t-tests or chi-square statistics. For the main analysis, random effects meta-analysis with logistic regression was used to evaluate the association between sexual orientation identity categories, mental health and wellbeing, adjusting for covariates. In preliminary analyses, we found that the proportion of variance in the effects accounted for by between-study variation ranged from 0 % to 53 % (as a proportion of total study variance), leading us to use random effects meta-analysis to pool study-specific odds ratios and their standard errors. Results were obtained first adjusted for age and sex, then after additional adjustment for covariates. We also examined whether sex, age and educational attainment significantly modified the association between sexual orientation identity groups and mental health, to identity population subgroups with higher relative risks of poor mental health. In sensitivity analyses to evaluate several possible sources of bias, we checked whether results differed materially when using the alternative ‘one stage’ approach to individual participant meta-analysis [32]. This involves analysing all data simultaneously with a random effect for study, rather than pooling results from each study separately. We also evaluated whether results differed when using only the GHQ-12 not the EQ5D, in case differences between these instruments influenced the results. Because some studies used over-sampling of some population groups and had other complex survey design features, we compared results separately for Understanding Society (the study with the largest contributing sample size) before and after accounting for the survey design, using sampling weights. We also reran models replacing the covariate ‘married or co-habiting’ with ‘married or civil partnered’ to check whether these affected the estimates in the fully adjusted model. All analyses were conducted using Stata version 13.1.

### Ethical approval

For all of the original studies used, ethical approval was provided by a university or local research ethics committee (see UK Data Service for details for each study). Written informed consent was provided by all participants. All data are available from the UK Data Service.

## Results and discussion

Of the 94,818 participants in the analytic sample (those with available data on sexual orientation identity, mental health and covariates), 97.2 % as heterosexual, 1.1 % identified as lesbian/gay, 0.9 % as bisexual and 0.8 % as ‘other’ (Table 1). People meeting the threshold of common mental disorder or low wellbeing were significantly different across all study variables (using bivariate *t*-test or chi-square tests): they were younger, comprised more females, and had lower levels of educational attainment, more current smokers, more longstanding illness/disability and fewer married/co-habiting participants than those below the threshold (Table 2). Significantly higher proportions of those who identified as lesbian/gay, bisexual and ‘other’ were found among those who met the mental disorder threshold.

**Table 2:** Characteristics of study variables comparing according to mental health and wellbeing

	Symptoms of common mental disorder				Anxious or depressed				Low wellbeing score			
	(GHQ-12)				(EQ5D)				(WEMWBS)			
	Yes	No	p	Total	Yes	No	p	Total	Yes	No	p	Total
	(*n* = 12,462)	(*n* = 60,401)		(*n* = 72,863)	(*n* = 3,251)	(*n* = 10,753)		(*n* = 14,004)	(*n* = 15,127)	(*n* = 42,029)		(*n* = 57,156)
Age (25/50/75 percentile)	31/46/62	27/43/57		30/46/61	34/48/63	37/50/63		35/48/63	38/46/62	40/46/60		38/46/61
Male (%)	35.7	45.8	<0.001	44.0	46.6	38.3	<0.001	44.7	42.6	44.7	<0.001	44.1
Lesbian/Gay (%)	1.6	0.9	<0.001	1.0	1.2	0.9	0.10	1.0	1.2	1.0	0.07	1.0
Bisexual (%)	1.9	0.6	<0.001	1.0	1.1	0.5	<0.001	0.7	1.0	0.6	<0.001	0.7
‘Other’ (%)	1.2	0.5	<0.001	0.9	0.8	0.3	<0.001	0.5	1.2	0.6	<0.001	0.7
Ethnic minority (%)	12.7	8.9	<0.001	9.5	8.6	9.6	0.08	9.4	3.8	5.0	<0.001	4.7
University degree (%)	22.6	26.5	<0.001	25.8	27.3	20.5	<0.001	25.7	18.9	30.5	<0.001	27.5
Smoker (%)	42.6	40.5	<0.001	40.9	26.7	17.1	<0.001	19.3	31.6	28.9	<0.001	29.7
Longstanding illness (%)	51.9	32.5	<0.001	35.8	62.7	35.4	<0.001	41.7	53.1	33.4	<0.001	38.6
Married/cohabiting (%)	51.3	63.6	<0.001	61.5	55.7	66.8	<0.001	64.3	56.2	69.4	<0.001	65.9

Compared to heterosexuals, participants identifying as lesbian/gay were more likely to have poor mental health, were significantly younger, comprised more men, fewer ethnic minorities, higher levels of educational attainment, more smokers, and fewer who were married or cohabiting (Table 3). Compared to heterosexuals, participants identifying as bisexual had similar patterns to lesbian/gay participants except no significant differences were found for sex or educational attainment. Additionally, there was a significantly higher proportion with longstanding illness/disability among bisexual participants compared to heterosexual participants. Participants identifying as ‘other’ were significantly different across all study variables compared to heterosexuals, except for the proportion of smokers which was similar.

Across each of the 12 surveys, the proportion of participants identifying as lesbian/gay ranged from 0.7 to 1.9 %, bisexual ranged from 0.5 to 1.7 %, ‘other’ from 0.2 to 1.4 %. Table 1 shows the sample size that each study contributed to the study, and differences across studies for study variables, including the refusal rate for the question about sexual orientation identity.

There was evidence that effects differed for men/women (p for interaction = 0.02) and by age group (p for interaction < 0.001) but not for ethnic minority status (p for interaction = 0.30) or educational attainment (*p*  = 0.19). Differences for men/women generally showed stronger effects for men but in the same direction for men and women. Differences across age groups were more pronounced, leading us to separate age groups for the main analysis and show men/women separately in Additional file 1: Table S1.

Results from the main pooled analysis are shown in Table 3. In the under 35 age group, lesbian/gay identity was associated with increased risk of symptoms of common mental disorder, adjusting for a range of covariates (OR = 2.06, 95 % CI 1.60, 2.66) when compared to heterosexuals in the same age group. The association was not significant in the 35–54.9 age group (OR = 1.03, 95 % CI 0.71, 1.48). The direction of the effect was consistent with a small increase in risk, but there was insufficient statistical power in this subgroup to estimate this effect with confidence. In the age group 55+ however, lesbian/gay identity was associated with more than twice the risk (OR = 2.11, 95 % CI 1.16, 3.83) of these symptoms than the heterosexual reference group. Patterns were similar in relation to low wellbeing, as measured by the WEMWBS, with the association weaker at midlife.

**Table 3:** Characteristics of participants identified as lesbian/gay, bisexual and ‘other’ compared to heterosexuals

	Heterosexual	Lesbian/Gay	p	Bisexual	p	‘Other’	p
Symptoms of common mental disorder (%)	16.8	26.2	<0.001	34.0	<0.001	23.8	<0.001
Anxious or depressed (%)	23.0	29.2	0.09	39.1	<0.001	43.9	<0.001
Low wellbeing score (%)	26.2	29.7	0.05	36.7	<0.001	44.2	<0.001
Age (25/50/75 percentile)	33/44/60	27/40/47	<0.001	20/34/50	<0.001	38/52/66	<0.001
Male (%)	44.1	60.1	<0.001	41.5	0.13	40.3	0.04
Ethnic minority (%)	9.0	5.7	<0.001	11.7	0.01	19.2	<0.001
University degree (%)	25.6	38.9	<0.001	27.2	0.28	11.8	<0.001
Smoker (%)	36.2	42.4	<0.001	41.8	0.001	38.2	0.26
Longstanding illness (%)	35.2	35.7	0.71	39.9	0.005	44.1	<0.001
Married/cohabiting (%)	63.0	40.0	<0.001	45.2	<0.001	54.9	<0.001

*Note. p* value for comparison with heterosexual group

Bisexual identity was associated with increased risk of poor mental health symptoms when compared to heterosexuals, across all age groups, with a similar pattern of effect modification: in the under 35 age group (OR = 2.31, 95 % CI 1.83, 2.90), lowest at age 35 to 54.9 (OR = 1.80, 95 % CI 1.29, 2.50) and strongest at age 55+ (2.45, 95 % CI 1.58, 3.79), adjusting for a range of covariates in relation to symptoms of common mental disorder. Patterns were broadly similar for low wellbeing, with the association weakest at midlife.

The group who identified as ‘other’ showed smaller effect sizes with wider confidence intervals, but the pattern was consistent with an increase in risk of meeting the threshold for disordered symptoms in all three groups when compared to heterosexuals in each age group: under 35 (OR = 1.96, 0.94, 4.09), 35–54.9 (OR = 1.63, 95 % CI 0.93, 2.86), age 55+ (OR = 1.27, 95 % CI 0.87, 1.86). Statistical power was not sufficient to estimate these smaller effects confidently, because of the limited sample size in these subgroups. This group were more likely than heterosexuals to have low wellbeing, across all three age groups, with weaker effects seen in older adults.

In sensitivity analyses, the pattern of results was the same after using the ‘one stage’ approach to analyse the pooled data. We also reran the models after excluding studies using the EQ5D rather than the GHQ-12. The results were not materially different, with lowest relative risks seen at midlife and highest in older adults. We also reran models for the ‘Understanding Society’ cohort after adjusting for the complex survey design using sampling weights. The same pattern of results was seen. Results were not materially different when adjusting for ‘married or civil partnered’ instead of ‘married or co-habiting’.

By pooling data from 12 population health surveys, we were able to show that lesbian, gay, bisexual and ‘other’ identified adults (non-heterosexual) were around twice as likely to report symptoms of poor mental health (i.e. anxiety, depression) than heterosexual adults. This result was less strong in female participants (see Table 4). The lowest relative risks were seen at midlife, with increased risk strongest in young non-heterosexual adults and highest for older non-heterosexual adults. Overall, bisexual (vs. heterosexual) adults had the highest risk of meeting the threshold for disordered symptoms.

**Table 4:** Odds ratios (95 % confidence intervals) for poor mental health and low wellbeing by sexual orientation identity across age groups

	Age <35		Age 35 to 54		Age 55+	
GHQ-12 score ≥4 or EQ5D anxious/depressed	Minimally adjusted^a^	Additionally adjusted^a^	Minimally adjusted^b^	Additionally adjusted^a^	Minimally adjusted^b^	Additionally adjusted^b^
Total *n* = 80,129	(*n* = 24,228)		(*n* = 27,520)		(*n* = 28,381)	
Lesbian/gay	1.92	1.78	1.75	1.42	2.24	2.06
(*n* =684)	(1.52, 2.43)	(1.40, 2.26)	(1.37, 2.24)	(1.10, 1.84)	(1.43, 3.52)	(1.29, 3.31)
Bisexual	2.52	2.15	2.38	1.88	2.34	2.47
(*n* = 829)	(2.04, 3.10)	(1.74, 2.67)	(1.73, 3.28)	(1.34, 2.65)	(1.64, 3.35)	(1.71, 3.58)
‘Other’	1.66	1.48	2.03	1.68	1.35	1.26
(*n* = 743)	(1.22, 2.45)	(0.99, 2.07)	(1.52, 2.71)	(1.24, 2.27)	(1.00, 1.82)	(0.93, 1.73)
Heterosexual	Reference	Reference	Reference	Reference	Reference	Reference
(*n* = 77,863)						
WEMWBS low wellbeing score	Minimally adjusted	Additionally adjusted	Minimally adjusted	Additionally adjusted	Minimally adjusted	Additionally adjusted
Total *n* = 57,156	(*n* = 11,170)		(*n* = 25,767)		(*n* = 20,219)	
Lesbian/gay	1.71	1.53	1.01	0.83	1.52	1.36
(*n* = 592)	(1.24, 2.37)	(1.09, 2.14)	(0.79, 1.29)	(0.65, 1.07)	(0.87, 2.65)	(0.77, 2.41)
Bisexual	2.79	2.45	1.25	0.98	1.18	1.23
(*n* = 425)	(2.00, 3.89)	(1.73, 3.47)	(0.88, 1.79)	(0.67, 1.42)	(0.79, 1.77)	(0.81, 1.87)
‘Other’	3.82	2.87	2.57	1.73	1.84	1.66
(*n* = 421)	(1.84, 7.93)	(1.35, 6.11)	(1.80, 3.67)	(1.19, 2.51)	(1.41, 2.41)	(1.26, 2.21)
Heterosexual	Reference	Reference	Reference	Reference	Reference	Reference
(*n* = 55,718)						

*Note*. ^a^ = minimally adjusted for age and sex ^b^ = additionally adjusted for ethnic minority status, educational attainment, cigarette smoking, longstanding illness/disability and relationship status. Total n refers to analytic sample with available data on sexual orientation identity, mental health or wellbeing, and covariates

This study is the first to pool sexual orientation identity data from 12 surveys, with data collected in the United Kingdom, using individual participant meta-analysis to determine the association with mental health (common mental disorder and wellbeing) symptoms. This approach provides sufficient power to examine subgroups, which is often not possible within each study because of low numbers. We were able to evaluate whether the association differed for men/women, across levels of educational attainment, for ethnic minorities and across the age range. The data contained a heterosexual comparison group, often not available in convenience samples. A standardised question was used to record sexual orientation identity, allowing comparability across studies. An important finding was that a number of participants selected ‘other’ but not ‘heterosexual’. It is not clear what participants intended in making this choice. It could reflect lack of understanding or literacy problems, a reluctance or refusal to be categorised by any of the more specific options, or self-identification as an identity not included in the list. It is also worth noting that this group contained the highest proportion of ethnic minorities, high levels of longstanding illness/disability and tended to be older. Future health surveys could collect additional detail on sexual orientation identity in order to clarify what this category means to participants.

The main limitation of our study was that results do not generalise beyond sexual orientation identity. Results may have differed if sexual orientation groups were defined in terms of sexual behaviour or sexual attraction, because adults with same-sex behaviour or same-sex attraction do not necessarily identify as non-heterosexual [2, 33]. When separating age groups, our models had statistical power >80 % to detect odds ratios larger than 1.5 (assuming 1 % in a comparison group and 99 % in a heterosexual comparison group, a sample size of 28,000, R-square of 0.10 and *p* = 0.05), but did not have sufficient statistical power to detect smaller effect sizes such as those seen in the ‘other’ group. A further limitation is that the question did not ask about change in identity over time. Sexual orientation identity can change over time, and change in sexual identity might also impact on mental health [34]. We did not consider longitudinal changes in mental health over time [35]. Although we considered age, sex, ethnic minority status and educational attainment as possible effect modifiers of the association between sexual orientation identity and mental disorder symptoms, further work could explore regional differences, as well as people with disabilities and other groups in the non-heterosexual population who might be more vulnerable than others. Given clear evidence of heterogeneity in the refusal rate for the question asked about sexual orientation identity (Table 1), there is a need to evaluate methodological differences across studies and the potential for bias according to mode of survey administration (e.g. face to face interview, telephone interview, self-completion questionnaire, web survey). There were 54 subgroups comparisons tested (Table 4 and Additional file 1: Table S1). We would therefore expect around three tests to be significant at *p* = 0.05 by chance. Statistical power was sufficiently high for evaluating the larger effect sizes observed here but not smaller effects including those seen for the ‘other’ group. It is important to note however, that all the subgroups we considered are important from a public health perspective in order to allocate resources and target services to subgroups of the LGB adult population who have different service needs [18, 19]. Our analysis was cross-sectional rather than longitudinal, meaning that we considered prevalence of poor mental health or low wellbeing, but not incidence. Elevated prevalence for a specific subgroup could be a function of higher incidence or longer duration of illness. Finally, the EQ-5D provides a very limited measure of mental disorder, comprising only one question on psychological symptoms that conflates anxiety with depression. Results were similar however, when excluding studies using this measure.

Our results are consistent with evidence internationally [9, 11–13] that non-heterosexual adults are at increased risk of mental health symptoms compared to heterosexuals, but provide important new insights by suggesting that younger and older non-heterosexual adults are particularly vulnerable (compared to those at mid-life). The cross-sectional nature of the data however, means that we cannot determine if these are aging, period, or cohort (generational) effects. These findings could reflect an existing observation that susceptibility to poor mental health is reduced in older adults [22], which may offer individual non-heterosexual adults some advantage in comparison to their younger peers.

Our study did not evaluate explanations for the associations between sexual orientation identity and mental health, that is, mechanisms or mediating variables. Mechanisms underlying an association between LGB orientation and poor mental health outcomes are not understood fully, but it has been argued that it is the experience of discriminatory and stigmatised experiences that can lead to increased mental disorder, as might early exposure to adversity [11]. Minority stress theory [36] suggests that internal and external manifestations of prejudice, victimization, and discrimination create observed health differences because these experiences are internalised. Chronic stress brought about by internalising stigma may therefore lead people who identify as non-heterosexual to experience poorer mental health and wellbeing [37, 38]; unhealthy behaviours [5] and worse physical health [4]. Certainly in LGB youth, evidence points to an increased risk of harassment and victimisation compared to heterosexual youth [39] and that the negative impact can be ameliorated by positive attitudes [40] and family support [41]. Many LGB adults do not disclose their sexual orientation to healthcare professionals, which could delay access to treatment [42, 43]. This study reinforces the need for clinicians to ensure that they provide services in which LGB patients can disclose their sexual orientation and receive supportive and integrated care.

Public health policies to address health inequalities require an evidence base that clarifies the extent of the problem. Population data on sexual orientation identity, which will provide policy makers and commissioners with the evidence they need, have only recently become available in the United Kingdom a limited number of data sets. Sexual orientation needs to become a part of routine data collection so that inequalities in poor mental health can be more fully understood. This study emphasises the need for continued, and expanded, collection of sexual orientation in all large health surveys and cohort studies to understand better the life course risks and impacts on outcomes for this population group. The cross-sectional data used in this study allows us to determine prevalence of poor mental health in this population. Future research is needed to determine whether these patterns track over time in longitudinal data. Longitudinal data will also allow us to monitor the incidence of new mental health problems rather than prevalence of existing symptoms, which might vary in duration. Further research is needed to consider what the underlying mechanisms of these associations are, and how interventions can be designed that remove inequalities in mental health between adults who identify as heterosexual and those who identity as lesbian, gay, bisexual or 'other'.

## Conclusions

- Adults identifying as lesbian, gay, bisexual or ‘other’ are at increased risk of poor mental health and low wellbeing compared to those identifying as heterosexual.
- The association varies across the life course, with the lowest relative risks seen in midlife and the highest among older adults
- Our study used cross-sectional data suitable for estimating prevalence, but future studies should consider longitudinal patterns (such as onset and persistence of new mental health problems) and clarify mechanisms

## Additional file

Additional file 1: Table S1. Odds ratios (95 % confidence intervals) for poor mental health by sexual orientation identity for men and women. (DOCX 20 kb)

## Abbreviations

BCS: British Cohort Study
EQ5D: EuroQoL version 5D
WEMWBS: Warwick-Edinburgh Mental Well-Being Scale
HSE: Health Survey for England
IAPT: Increased Access to Psychological Therapies service
NHS: National Health Service
LGB: lesbian, gay or bisexual
LSYPE: Longitudinal Study of Young People in England
ONS: Office of National Statistics
SHS: Scottish Health Survey
UK: United Kingdom
US: Understanding Society
