# Supplementary material for: Sexual orientation and symptoms of common mental disorder or low wellbeing: combined meta-analysis of 12 UK population health surveys
Source: BMC Psychiatry. 2016 Mar 24;16:67. doi: 10.1186/s12888-016-0767-z (PMC4806482; doi:10.1186/s12888-016-0767-z)
Supplement: Additional file 1: Table S1. — Odds ratios (95 % confidence intervals) for poor mental health by sexual orientation identity for men and women. (DOCX 20 kb) [file 12888_2016_767_MOESM1_ESM.docx]

Supplementary Table 1. Odds ratios (95% confidence intervals) for poor mental health by sexual orientation identity for men and women

|  | Women | | Men | | Total | |
| --- | --- | --- | --- | --- | --- | --- |
| GHQ-12 score ≥4 or EQ5D anxious/depressed | Minimally adjusted | Additionally adjusted | Minimally adjusted | Additionally adjusted | Minimally adjusted | Additionally adjusted |
|  | (n = 44,807) | | (n = 35,322) | | (n = 80,129) | |
| Lesbian/gay (n =684) | 1.38 (1.07, 1.78) | 1.18 (0.91, 1.53) | 2.25 (1.84, 2.75) | 2.05 (1.66, 2.53) | 1.82 (1.56, 2.13) | 1.62 (1.38, 1.91) |
| Bisexual (n = 829) | 2.23 (1.83, 2.73) | 1.89 (1.53, 2.34) | 2.66 (2.08, 3.42) | 2.24 (1.24, 1.73) | 2.37  (2.03, 2.76) | 2.03 (1.72, 2.38) |
| ‘Other’ (n = 743) | 1.69 (1.36, 2.11) | 1.54 (1.23, 1.93) | 1.52 (1.10, 2.07) | 1.28 (0.92, 1.76) | 1.59 (1.33, 1.90) | 1.42 (1.18, 1.70) |
| Heterosexual (n = 77,863) | Reference | Reference | Reference | Reference | Reference | Reference |

*Note*. ^a^=minimally adjusted for age and sex ^b^ = additionally adjusted for ethnic minority status, educational attainment, cigarette smoking, longstanding illness/disability and relationship status.
